# Signaling C-type lectin receptors in antimycobacterial immunity

**DOI:** 10.1371/journal.ppat.1006333

**Published:** 2017-06-22

**Authors:** Mohlopheni J. Marakalala, Hlumani Ndlovu

**Affiliations:** 1 Division of Immunology, Department of Pathology, Institute of Infectious Disease and Molecular Medicine, University of Cape Town, Observatory, South Africa; 2 Department of Integrative Biomedical Sciences, Division of Chemical and Systems Biology, University of Cape Town, Observatory, South Africa; Tufts Univ School of Medicine, UNITED STATES

## Introduction

**Fig 1:**
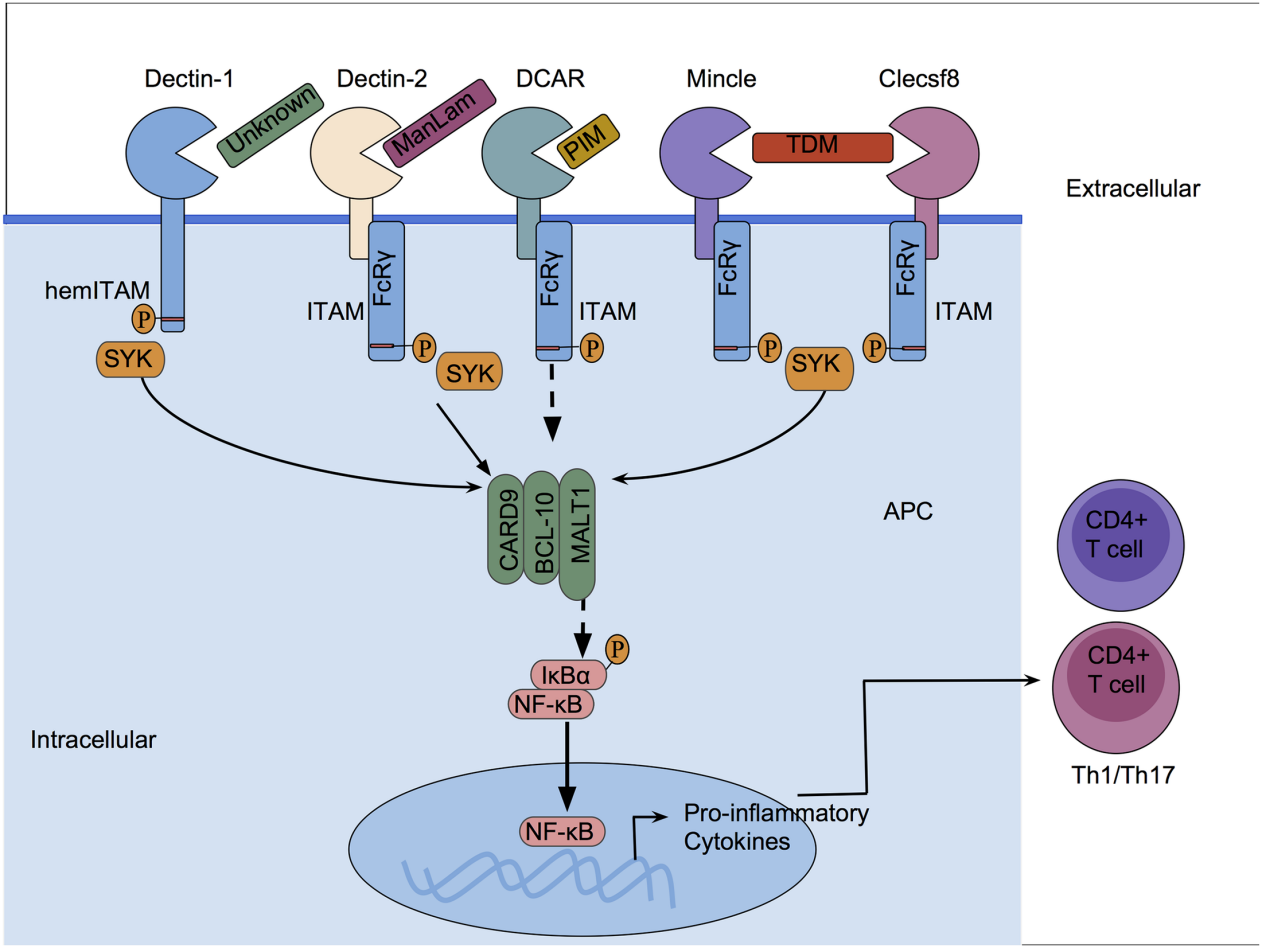
Recognition of mycobacterial pathogen-associated molecular patterns (PAMPs) by C-type lectin receptors (CLRs). Dectin-2 recognizes mannosylated lipoarabinomannan (ManLAM), dendritic cell immunoactivating receptor (DCAR) recognizes phosphatidylinositol mannosides (PIMs), macrophage-inducible C-type lectin (Mincle) and C-type lectin superfamily member 8 (Clecsf8) recognize the glycolipid trehalose 6,6’ dimycolate (TDM), while the mycobacterial ligand of Dectin-1 is yet to be identified. The interaction of the CLRs with mycobacterial PAMPs triggers cytoplasmic signaling and a number of cellular responses. The CLRs signal via Spleen tyrosine kinase (Syk), which associates with the Caspase recruitment domain family member 9 (CARD9)/B-Cell CLL/lymphoma 10 (BCL-10)/Mucosa-associated lymphoid tissue lymphoma translocation protein 1 (MALT1) complex, resulting in the production of pro-inflammatory cytokines and induction of adaptive T-cell immunity.

The mammalian innate immune system is composed of phagocytes such as macrophages and dendritic cells that serve as the first line of defense against microbial infections. These cells express various pattern recognition receptors (PRRs) that recognize specific pathogen-associated molecular patterns (PAMPs) on the surface of or inside microorganisms [1]. PRRs such as Toll-like receptors (TLRs), C-type lectin receptors (CLRs), and Nucleotide-binding Oligomerization Domain (NOD)-like receptors (NLRs) have been widely studied in antimicrobial immunity and homeostasis. These PRRs have also been implicated in antimycobacterial immunity, with CLRs recently receiving considerable attention. CLRs are a large family of proteins containing at least 1 carbohydrate-recognition domain (CRD) that in most cases binds a range of carbohydrate-based PAMPs, including trehalose 6,6’ dimycolate (TDM), lipoarabinomannan (LAM), lipomannan (LM), and phosphatidylinositol mannosides (PIMs) [2–4]. Interactions of CLRs with mycobacterial PAMPs induce intracellular signaling that triggers responses ranging from cytokine production to induction of adaptive immunity (Table 1). Here, we discuss signaling CLRs that recognize mycobacterial PAMPs and contribute to antimycobacterial immunity. We focus on the receptors that signal through the Spleen tyrosine kinase (Syk)/Caspase recruitment domain family member 9 (CARD9) pathway, including Dectin-1, Dectin-2, macrophage-inducible C-type lectin (Mincle), C-type lectin superfamily member 8 (Clecsf8) also called macrophage C-type lectin (MCL), and dendritic cell immunoactivating receptor (DCAR) (Fig 1).

**Table 1:** C-type lectin receptors, mycobacterial ligands, and their effects on pro-inflammatory cytokine production and contributions in host resistance to mycobacterial infections in vivo.

C-type lectin receptor	Mtb ligand	Cellular expression	Effects on pro-inflammatory cytokine production	Role in host resistance to mycobacterial infection	References
**Dectin-1**	unknown	DCs, monocytes, macrophages, neutrophils, eosinophils, mast cells, and lung epithelium	↑IL-6, IL-23, IL-1β, TNF-α, IL-12p40, and IL-17	Dispensable for host resistance to *Mycobacterium tuberculosis* H37Rv infection in mice	[5, 7, 9–11, 35]
**Dectin-2**	ManLAM	DCs, monocytes, tissue macrophages, CD8^+^ T cells, and CD19^+^ B cells	↑TNF-α, IL-6, and IL-17	Survival studies not performed. Required to control lung damage during *M*. *avium* infection.	[4, 12, 14, 36]
**Mincle**	TDM	Monocytes, macrophages, neutrophils, myeloid DCs, and B cells.	↑IL-8, IL-6 andIL-1β	Required for bacterial clearance. Inconsistent results on essentiality.	[4, 17, 19, 21–23]
**ClecSF8 (MCL)**	TDM	Neutrophils, monocytes, and DCs	↑IL-6, TNF-α and IL-1β	Required for resistance to *M*. *bovis* BCG and *M*. *tuberculosis* H37Rv infection in mice	[25, 28, 29]
**Mannose receptor**	ManLAM, DIM, mannosylated proteins	Macrophages and MDCs	↑IFN-γ	Survival studies not performed	[10, 34, 37, 38]
**DC-SIGN**	ManLAM, PIMs, mannosylated glycoproteins	Myeloid DCs and macrophages	↑IFN-γ	hSIGN transgenic mice resistant to high-dose *M*. *tuberculosis* H37Rv infection. SIGNR3 KO mice have elevated CFUs.	[10, 32, 33, 39]
**DCAR**	PIMs	Peritoneal macrophages, monocyte-derived inflammatory cells in lung and spleen	↑IFN-γ and IL-12	Survival studies not performed. High CFU in DCAR KO mice infected with BCG or H37Rv.	[4, 31]

**Abbreviations**: CFU, colony-forming unit; ClecSF8, C-type lectin superfamily member 8; DC, dendritic cells; DC-SIGN, Dendritic Cell-Specific Intercellular adhesion molecule-3-Grabbing Non-integrin; DCAR, dendritic cell immunoactivating receptor; DIM, Phthiol Dimycocerosates; KO, knock-out; ManLAM, Mannose-caped Lipoarabinomannan; Mincle, macrophage-inducible C-type lectin; MCL, macrophage C-type lectin; Mtb, Mycobacterium tuberculosis; PIM, Phosphatidyinositol Mannosides; TDM, Trehalose Dimycolate.

## Dectin-1

Dectin-1 is a glycosylated transmembrane receptor possessing an extracellular C-type lectin-like domain (CRD) and a cytoplasmic immunoreceptor tyrosine-based activation motif (ITAM)-like domain, also known as hemITAM, which initiates downstream signaling and cellular activation [5]. This archetypical CLR has been extensively characterized as a major fungal β-1,3-glucan receptor that can mediate various immune responses, including phagocytosis, respiratory burst, cytokine and chemokine production, and direct instruction of Type 1 T-helper (Th1) and Type 17 T-helper (Th17) immunity [5]. Dectin-1 is predominantly expressed on macrophages, dendritic cells, neutrophils, and a subset of T cells. Consistent with its potential role in immune surveillance, Dectin-1 is highly expressed in portals of pathogen entry, including the intestines and the lung [5]. A number of studies have associated Dectin-1 with a role in antimycobacterial immunity, although its mycobacterial ligand remains unknown. Dectin-1 promotes production of IL-6, G-CSF, and RANTES in bone marrow—derived macrophages stimulated with attenuated *Mycobacterium bovis* (*M*. *bovis*) and avirulent H37Ra *Mycobacterium tuberculosis* (Mtb) strain [6]. In splenic dendritic cells (DCs) infected with *M*. *bovis* or pathogenic Mtb, Dectin-1 triggers IL-12p40 production in a Syk-dependent manner [7]. Dectin-1 has also been demonstrated to cooperate with TLR2 for efficient uptake of *M*. *abscessus* by murine macrophages and subsequent induction of pro-inflammatory cytokines [8]. The cooperation between the 2 receptors has also been reported in human A549 airway epithelial cells infected with Mtb [9]. Further studies on human cells have shown that stimulation of monocyte-derived DCs with Mtb leads to Dectin-1–dependent production of pro-inflammatory responses, facilitating the DCs to instruct T cells to produce IFN-γ and IL-17 [10]. However, Dectin-1–deficient mice are resistant to Mtb infection, similarly to wild-type mice, despite slightly reduced lung bacterial burdens [11]. Thus, Dectin-1 seems to play a redundant role in antimycobacterial defense in vivo, despite inducing impressive pro-inflammatory cytokine responses in vitro. There is currently no known association of human Dectin-1 polymorphisms with Tuberculosis (TB) disease susceptibility.

## Dectin-2

Dectin-2 structure is made up of a CRD, a transmembrane domain, and a short cytoplasmic tail. Although Dectin-2 expression was originally proposed to be specific to Langerhans cells, subsequent work has demonstrated that this PRR is predominantly expressed on myeloid cells, including tissue macrophages, some subsets of dendritic cells, and peripheral blood monocytes, in which its expression can be up-regulated by various inflammatory stimuli [12,13]. Dectin-2 specifically recognizes mycobacterial mannosylated lipoarabinomannan (ManLAM), resulting in a cascade of downstream signaling and cellular activation [13,14]. Unlike Dectin-1, the short cytoplasmic tail of Dectin-2 does not contain an ITAM-like motif. Instead, it recruits an ITAM-linked FcRγ, which initiates signaling through Syk and likely the Caspase recruitment domain family member 9/B-Cell CLL/lymphoma 10/Mucosa-associated lymphoid tissue lymphoma translocation protein 1 (CARD9/BCL10/MALT1) complex [13]. Dectin-2 induces production of pro- and anti-inflammatory cytokines, including IL-10, IL-2, IL-6, MIP-2, and TNF after stimulation of DCs with ManLAM and BCG [14]. The Dectin-2–ManLAM interaction also induces T-cell responses. Dectin-2 deficiency results in enhanced pathology in mice infected with nontuberculous *M*. *avium* [14]. Dectin-2 has been demonstrated to recognize the virulent Mtb H37Rv strain [14]; however, the protective role of this receptor against Mtb has not been shown in vivo.

## Mincle

Mincle is predominantly expressed on cells of myeloid lineage, including macrophages, neutrophils, and DCs, as well as other cell types, such as B cells and microglia in the brain [15]. Mincle structure is composed of a CRD, a transmembrane domain, and a short cytoplasmic tail with a positively charged residue, through which it associates with the adaptor molecule FcRγ and initiates intracellular signaling via the Syk/CARD9 pathway [16, 17]. The major mycobacterial ligand for Mincle is TDM (also known as the cord factor), the most abundant mycobacterial cell wall glycolipid [18,19]. Deletion of Mincle results in impaired production of pro-inflammatory cytokines and nitric oxide by macrophages after stimulation with TDM or its synthetic analog, trehalose 6,6-dibehenate (TDB). Moreover, treatment of mice deficient of Mincle with TDM results in significantly reduced TNF-α and IL-6 production. Mincle can also trigger robust Th1 and Th17 immunity in mice treated with TDB as an adjuvant to H1 subunit vaccine (an Mtb fusion protein of antigen 85 B [Ag85B] and the 6kDa early secreted antigenic target [ESAT-6]) [18–20]. Mincle signaling on neutrophils has been demonstrated to drive TDM-induced lung inflammation and promote cell adherence by enhancing F-actin polymerization and CD11b/CD18 surface expression. These Mincle-driven responses are dependent on Src, Syk, and mitogen-activated protein kinases (MAPK)/extracellular-signal-regulated kinase (ERK) kinases and can also be augmented by TLR2 coactivation [21]. Mincle requirement in the control of TB in vivo remains unclear, with some contradictory findings [4,17]. A study by Lee et al. showed that Mincle deficiency results in elevated bacterial burdens in the lungs of mice infected with Mtb [21]. The requirement of Mincle for bacterial clearance has also been demonstrated in mice infected with *M*. *bovis* BCG [22]. Another study, however, has demonstrated that the receptor is dispensable for Mtb control, with Mincle-deficient mice mounting the same immune response as wild-type mice, with similar T-helper immunity, lung bacterial burdens, and macrophage effector mechanisms [23]. A recent report has also demonstrated that Mincle is not associated with TB disease susceptibility or protection in humans [24]. The redundancy of Mincle in humans and possibly in mice is still poorly understood, but it is possible that other receptors that engage the same signaling pathway may compensate for the loss of Mincle. One such candidate is Clecsf8, which can engage the same mycobacterial ligand and trigger the same intracellular signaling pathway as Mincle. The next section will focus on Clecsf8 and its potential cooperation with Mincle.

## Clecsf8 (MCL or Clec4d)

Clecsf8 is an endocytic receptor that interacts with mycobacteria through the cell-wall glycolipid, TDM. This PRR is predominantly expressed on macrophages, peripheral blood neutrophils, classical monocytes, and some subsets of DCs. Upon engagement of TDM, Clecsf8 positively regulates Mincle expression through a protein—protein interaction, resulting in augmented cellular responses [25,26]. Downstream signaling of this FcRγ-coupled receptor is mediated through Syk kinase and CARD9/BCL10/MALT1 pathways and induces various intracellular responses, including NFκβ activation, pro-inflammatory cytokine production, phagocytosis, and respiratory burst. Clecsf8 interaction with TDM also induces DC maturation and T-cell priming [26–28]. Loss of this receptor in mice results in increased susceptibility to Mtb infection, with disease phenotypes characterized by high bacterial loads in the lung, augmented inflammatory responses, increased pathological damage accompanied by neutrophilic infiltration, and early mortality [29]. Thus, Clecsf8 seems to be essential for TB control in vivo. However, disease effects associated with Clecsf8 deficiency are only a fraction compared to the susceptibility associated with the loss of the central downstream adaptor, CARD9 [30]. This suggests some level of compensation by other receptors in addition to Clecsf8 that might be operating in collaboration to drive the CARD9-mediated antimycobacterial responses. The cooperation of Clecsf8 and other CLRs will be an interesting area of exploration in TB infection in vivo. In humans, polymorphisms of Clecsf8 are associated with TB susceptibility [29], making this CLR a key component of antimycobacterial defense.

## Other CLRs

DCAR is an FcRγ-coupled receptor that is predominantly expressed on monocyte-derived inflammatory cells and recognizes mycobacterial glycolipids called PIMs. DCAR-deficient mice have impaired IFNγ production by T cells and increased bacterial loads, indicating the importance of this receptor in the induction of antimycobacterial Th1 immune response [31]. Another extensively studied CLR is Dendritic Cell-Specific Intercellular adhesion molecule-3-Grabbing Non-integrin (DC-SIGN), which recognizes a number of mycobacterial ligands, including mannose-containing ManLAM and LMs. DC-SIGN recognition of mannose-containing PAMPs leads to a RAF-1 signalosome that induces cytokine production and promotes Th1 and Th17 differentiation [32]. A mouse homolog of DC-SIGN, SIGNR3, has been shown to recognize mycobacterial ManLAM, leading to production of IL-6 and TNF-α in a Syk-dependent manner [33]. Mannose Receptor (MR) also recognizes a number of mycobacterial mannose-containing PAMPs, including ManLAM, higher PIMs, LM, and other mannosylated proteins. MR is predominantly expressed on alveolar macrophages, and its interaction with ManLAM induces production of anti-inflammatory cytokines [34]. More work is still required in understanding downstream signaling of MR.

## Concluding remarks

Studies of CLR interaction with mycobacterial PAMPs have revealed novel insights into signaling mechanisms that drive antimycobacterial immunity. CLRs such as Mincle and Clecsf8 recognize mycobacterial glycolipids TDM and TDB and induce various innate immune responses and T-cell immunity. Recognition of ManLAM by Dectin-2 and DC-SIGN also induce pro-inflammatory cytokine production and T-cell responses. Thus, the immunomodulatory effects induced by these CLR—PAMPs interactions present an exciting area that can be explored for vaccine development. TDM and TDB have demonstrated great potential as adjuvants for H1 subunit vaccines, indicating a promising therapeutic potential for other mycobacterial ligands. Most of the CLRs discussed here signal via the Syk/CARD9 downstream pathway, which is essential for TB control. However, many of these CLRs seem to be individually dispensable in vivo, possibly due to compensation by other receptors. Such redundancies are still poorly understood and warrant further research. Moreover, there is a need to explore in detail the synergistic cooperation between the CLRs and other receptors such as TLRs and how this would affect the outcome of TB disease in vivo.
